# Validation of the Global Lung Function Initiative 2012 Spirometry Reference Values in a Healthy Italian Working Population

**DOI:** 10.3390/ijerph192215200

**Published:** 2022-11-17

**Authors:** Luca Fontana, Dante Luigi Cioffi, Veruscka Leso, Emanuele D’Ausilio, Daniela Pacella, Mauro Fedele, Mauro Maniscalco, Ivo Iavicoli

**Affiliations:** 1 Section of Occupational Medicine, Department of Public Health, University of Naples Federico II, Via S. Pansini 5, 80131 Naples, Italy; 2 Pulmonary Rehabilitation Unit, Institute ICS Maugeri SpA SB, Via Bagni Vecchi 1, 82037 Telese Terme, Italy

**Keywords:** GLI 2012, ERS 1993, reference values, pulmonary function test, workers, health surveillance

## Abstract

Background: Several studies showed important differences in the interpretation of spirometry based on different reference values, in particular by comparing European Respiratory Society (ERS) 1993 and Global Lung Function Initiative (GLI) 2012. The validation of new reference values in local populations is essential before they can be used in an appropriate manner. This study aimed to investigate the fit of GLI reference values in a healthy Italian working population. Methods: Spirometry data were collected in 1355 workers during their health surveillance medical examination conducted for exposure to chemical and biological risk factors. A single portable spirometer that met the ATS/ERS recommendations for occupational testing was used and calibrated daily. Results: Average z-score were −0.13 (with a median of −0.17), −0.25 (with a median of −0.24), and 0.18 (with a median of 0.17) for FEV_1_, FVC, and FEV_1_/FVC ratio, respectively. Considering only the normal-weighted workers, the average z-scores were −0.07 (with a median of −0.16), −0.15 (with a median of −0.16), and 0.07 (with a median of 0.02) for FEV_1_, FVC, and FEV_1_/FVC ratio, respectively. Conclusions: GLI 2012 reference values fit the Italian data satisfactorily, resulting as more accurate than ERS 1993, especially in women, normal-weighted subjects, aged 30−50 years, and for height < 165 cm.

## 1. Introduction

Spirometry is the main pulmonary function test (PFT) used to assess lung function and for this reason, in the field of Occupational Medicine (OM), it is fundamental for the diagnosis and prevention of occupational lung diseases [1]. In detail, simple spirometry, through the study of dynamic lung parameters and the forced expiratory maneuver, represents a key tool in the context of workers’ health surveillance to early identify ventilatory deficits that could influence the fitness for work-related to a specific job. Considering that lung function varies with age, gender, height and ethnicity, the Forced Vital Capacity (FVC) and Forced Expiratory Volume at 1st second (FEV_1_) obtained by simple spirometry are expressed as percentage of predicted values and the Lower Limit of Normal (LLN) is used for the diagnosis of lung impairment [2]. This means that they are compared with appropriate reference values obtained from healthy subjects with the same characteristics as those evaluated. The validation of the reference values is obtained by choosing a sample of subjects in good health and therefore, in the definition of the sample population, various exclusion criteria have to be applied. These are mainly based on the pathological history (presence of cough, bronchial hypersecretion, asthma, infectious pathologies, heart disease) or lifestyle habits (e.g., smoking) and if the values of the chosen sample approach the ideal theoretical value (100% of the predicted), the validation can be considered reliable [3,4].

Over the years, the reference equations proposed for European populations have been numerous, both through international and local studies. Until recently, the most accepted reference values used in clinical practice were those provided by the European Coal and Steel Community (ECSC) in 1983 [5], which were later confirmed by the European Respiratory Society (ERS) in 1993 [6]. This set of equations is still extensively used in Italy, although its use was advised by the statement on spirometry standardization published by the ERS and the American Thoracic Society (ATS) in 2005 [7]. In 2012, the Global Lung Initiative (GLI) produced a newer set of reference values calculated with advanced statistical methods and derived from a large number of healthy subjects aged from 3 to 95 years (57.395 Caucasians) from all over the world, including Italy (1818 Italian subjects were included in the final analyses) [8].

Noteworthy, together with the percentage of the predicted, they provided the z-score, a value that describe how many standard deviations a measured value differs from the predicted value, with only 5% of healthy subjects having a z-score of 1.64 or less [8]. The validation of these new reference values in a number of countries is very important for their correct use since the available literature data demonstrate how the use of different reference values (i.e., ERS 1993 vs. GLI 2012) can lead to rather significant interpretative differences [4]. Currently, the GLI 2012 have obtained international consensus and have been validated for their application in many countries, such as France, Germany, Norway, Australia, and Algeria [9,10,11,12,13], although in others they were considered not adaptable to local populations, such as in Tunisia, Finland, and Sweden, for which new, more suitable specific equations have been produced [14,15,16,17].

In detail, Hulo et al., showed that GLI 2012 equations fit better than those of ECSC to their French population sample and can be used for a French population aged 40–65 years [9]. In this regard, all z-score means were further from zero using the ECSC equations than when GLI equations were used [9]. Similarly, Langhammer et al., in 2016, observing that the median z-scores were respectively 0.02 ± 1.03, 0.01 ± 1.04, and −0.04 ± 0.91 for FEV_1_, FVC, and FEV_1_/FVC in males, and −0.01 ± 1.02, 0.07 ± 0.97, and −0.21 ± 0.82 in females, concluded that the GLI 2012 reference values fit the Norwegian data satisfactorily and so recommended them for use in Norway [11]. In this context, a real validation study of the GLI 2012 in the healthy working population has not yet been carried out in Italy. Therefore, our study aimed to validate the GLI 2012 reference values for spirometry in an Italian working population (that is determining whether these reference values are suitable for the specific studied population) in order to provide the Occupational Physicians (OPs) with scientific-based information that can support them in assessing the adoption of these new reference values in professional practice. Secondly, we compared the GLI 2012 reference values with the ERS 1993 (that are currently used) to highlight possible interpretative differences and give a clear indication to Italian OPs.

## 2. Materials and Methods

### 2.1. Study Population

The study data were obtained carrying out spirometric tests (January 2015–July 2019) during the health surveillance of workers exposed to several occupational risk factors including chemical and biological risk factors. All physicians involved in performing spirometric tests were trained at the same university and shared procedures for performing and interpreting tests. Since January 2017, all spirometries were performed by a single trained technician, supervised by the authors. Before carrying out the spirometry test, adequately trained medical staff collected an accurate personal, physiological, pathological, and occupational medical history in order to obtain information related to the job, smoking habit and any pathologies. In more detail, as far as the degree of obesity is concerned, we used the classification proposed by the World Health Organization, according to which a subject is considered obese when their body mass index (BMI) is ≥30.0 (obese class I: 30.00−34.99, obese class II: 35.00−39.99, and obese class III: ≥40.00). All spirometries were included regardless of age, and the anthropometric characteristics of the subjects undergoing health surveillance.

### 2.2. Pulmonary Function Test (Spirometry)

Spirometric tests were obtained from a single spirometer (a portable “Spirolab III” turbine flowmeter, MIR, Rome, Italy) used at the “Federico II” University Hospital of Naples (Italy) during the execution of health surveillance medical examinations. A factory-calibrated reusable turbine flowmeter was used, the turbine’s blades were regularly cleaned, and a daily calibration of the instrument, that has been tested to meet the ATS/ERS recommendations for occupational testing [1], was performed using a special pre-calibrated and certified 3 L syringe (0.5 L/s injection over 6 s) (MIR, Rome, Italy). All spirometric tests were performed according to the ATS/ERS 2019 criteria [18]. For the selection of spirometric tests to be included in the subsequent analyses, the following inclusion criteria were followed: (i) spirometry performed during the health surveillance medical examination, (ii) Caucasian race (to achieve the most homogenous investigated population), (iii) University workers exposed to chemical and/or biological risk factors, (iv) choice of the most recent exam in the case of multiple tests performed by the same worker, (v) compliance with the criteria of acceptability and repeatability of the tests according to the ATS/ERS 2019 standards [18], (vi) non-smoking habit, and (vii) absence of any pathological conditions that could have altered the test results.

### 2.3. Data Processing and Statistical Analyses

The data were processed using the free ERS software “GLI Excel sheet calculator” (software version 2.0, Global Lung Function Initiative, Lausanne, Switzerland) to obtain the spirometric values according to the GLI 2012 reference equations, the LLN and the z-scores, and were included into an Excel calculation file. For each spirometric test, the following information was collected: gender, age, height, weight, indications on the acceptability and reproducibility of the test, FVC (in L), FEV_1_ (in L), and FEV_1_/FVC (in %). FVC, FEV_1_, and FEV_1_/FVC were calculated on the basis of GLI 2012 equations. In our validation of the GLI 2012 for the Italian population, the approach of the z-score to 0 (100% of the theoretical) indicates a good reliability of the parameters measured as they are close to the ideal value, limiting possible cases of underestimation of the functional alterations assessed. In order to evaluate the presence of pathological tests, the criteria provided by the ATS/ERS 2005 guidelines and by the 2012 GLI reference values represented by the z-score were used (Obstructive deficit: FEV_1_/FVC ratio lower than the LLN or the z-score <−1.64; Restrictive pattern: FEV_1_/FVC ≥ LLN and FVC < LLN) [7,8].

The collected data were exported to the SPSS v 16.0 program for descriptive and comparative statistical analysis. The continuous variables were described as mean ± standard deviation and as median, indicating the minimum and maximum values; the dichotomous variables were expressed in absolute value and as a percentage of the total. The comparative analysis of FEV_1_, FVC, and FEV_1_/FVC, expressed as averages of the z-score and percentage of the predicted, was performed using the Mann–Whitney-U nonparametric test. For the comparison between age and height groups, the ANOVA test was used, followed by LSD post-hoc analysis for the comparison of the individual groups with each other. In all cases, a value of *p* < 0.05 was considered statistically significant. Finally, Bland–Altman plot and the intra-class correlation coefficient (ICC) were used to analyze the agreement between GLI 2012 and ERS 1993 reference values.

## 3. Results

From the 7450 starting spirometric tests, the 3642 tests performed in 2015 and 2016 were excluded because they were conducted by different trained physicians and this fact could have a very important impact on the accuracy and quality of the tests. From the 3808 resulting tests, 1268 multiple tests performed by the same worker in different years were excluded, choosing the most recent exam for the analysis. From the 2540 remaining tests, a final number of 1355 tests were obtained (53.4%). We excluded 545 (21.5%) pulmonary function tests because they did not meet the ATS/ERS 2019 criteria (in particular referring to lack of reproducibility). Furthermore, 476 (18.7%) smokers were excluded, 108 (4.2%) workers were not enrolled as they had a history of respiratory diseases, and finally 56 other tests (2.2%) were not included since they were performed by workers not subjected to health surveillance for exposure to chemical and/or biological risk factors.

The main socio-demographics characteristics of the 1355 workers whose spirometric tests were analyzed and the comparisons by gender are summarized in Table 1.

Considering all 1355 workers, the FEV_1_ mean value, expressed as a percentage with respect to the GLI 2012 reference equations, was 98.20%, with a median of 97.78% and a range between 73.07% and 132.65%. The percentage of predicted FVC mean value was 96.70%, with a median of 96.78%, and a range between 73.12% and 137.42%. With regard to the FEV_1_/FVC ratio, expressed as a percentage of the predicted using the GLI 2012, its mean value was 101.22%, with a median of 101.33%, and a range between 86.77% and 121.59% (Table 2). Analyzing the z-score, there was an average of the z-score regarding the FEV_1_ of −0.13, with a median of −0.17, in a range of values between −2.23 and 2.84. Considering the FVC, instead it had an average of the z-scores of −0.25, with a median of −0.24 in a range of values between −2.16 and 2.56. Concerning the FEV_1_/FVC ratio, the average of the z-scores was 0.18, with a median of 0.17 in a range between −1.63 and 3.17 (Table 2).

Using the parameters and classifications provided by the 2005 ATS/ERS standards and by the GLI working group, 1297 (95.7%) workers had a normal spirometry and when the GLI 2012 reference values were applied, no obstructions were detected and only 58 restrictive patterns (4.3%) were observed. It should be noted that, regarding the FEV_1_/FVC ratio, no spirometry of the 1355 workers showed a z-score lower than −1.64, which represents the fifth percentile and is therefore considered the LLN for obstructive deficit. In greater detail, considering only the 719 normal-weighted workers (i.e., having a BMI <25 kg/m^2^), the values tended even more evidently to 100% of the predicted GLI 2012 values and to 0 of the z-score (Table 2). Indeed, in this case there was an average of the z-score for FEV_1_ of −0.07, with a median of −0.16 and a range of values between −1.63 and 2.58, whereas the average FVC was −0.15, with a median of −0.16 and a range of values between −2.15 and 2.56 and the average FEV_1_/FVC ratio had a z-score of 0.07, with a median of 0.02, a minimum of −1.63, and a maximum of 2.58 (Table 2). The normal-weighted workers, compared with the obese ones, had a significantly greater proximity to 100% and zero z-score. By evaluating the z-score, the average FEV_1_ of non-obese subjects was significantly different and closer to 0 compared with obese subjects (−0.10 vs. −0.24, *p* = 0.008), significance which is achieved by comparing the mean values of FVC (−0.20 vs. −0.48, *p* < 0.001) and the FEV_1_/FVC ratio (0.14 vs. 0.40, *p* < 0.001).

Taking into account the gender, the mean values of FEV_1_, FVC, and FEV_1_/FVC obtained in the women sample, both as percentages of the predicted and as z-score, they were significantly closer to 100% than the men. In particular, there was a statistically significant difference in the z-score averages between men and women regarding FEV_1_ (−0.18 vs. −0.08, *p* = 0.026), FVC (−0, 34 vs. −0.16, *p* < 0.001), and FEV_1_/FVC ratio (0.29 vs. 0.08, *p* < 0.001). Moreover, according to the age, it was possible to divide the sample of 1355 workers into five groups of comparable numbers: 496 workers aged 18−30 years (36.6%), 212 aged 30−40 years (15.6%), 186 aged 40−50 years (13.7%), 247 aged 50−60 years (18.2%), and 214 aged 60−70 years (15.8%). Considering the z-score averages in these sub-groups, it was possible to notice that the middle ages had the greatest proximity to 0 for FEV_1_ (Table 3). On the other hand, with regard to the FVC, with increasing age a linear increase was observed in the distance from 0, with a z-score mean value of −0.16 in those 18−30 years and of −0.38 in workers over 60 years (Table 3). Similarly, for the FEV_1_/FVC ratio, the proximity to zero of the z-score average was maximum for the youngest, reaching −0.01 between 18 and 30 years (Table 3). Comparing these data using the ANOVA analysis for the various age groups, the difference appeared significant for FVC (*p* < 0.001) and FEV_1_/FVC (*p* < 0.001), but not for FEV_1_ (*p* = 0.226).

Depending on the height, the distinction was made into three comparable groups: 429 workers <165 cm tall (31.7%), 568 165−175 cm tall (41.9%), and the remaining 358 >175 cm tall (26.4%). In the case of FEV_1_, the shorter workers had an average z-score of −0.02, those at intermediate height of −0.17, and the taller workers of −0.19 (Table 4). For the FVC values, too, the average z-score was −0.13 for the shorter group, while for taller workers was −0.30 and −0.31. Considering the FEV_1_/FVC ratio, the averages are comparable in the three groups (Table 4).

**Figure 1:**
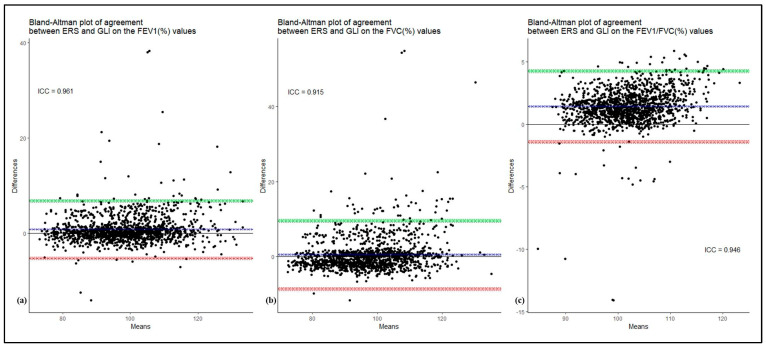
Bland–Altman plot and ICC for the comparison of FEV_1_% (**a**), FVC% (**b**), and FEV_1_/FVC values (**c**) using ERS 1993 and GLI 2012. The plots display the means (*x*-axis) and the differences (*y*-axis) between the two methods, along with the lower and upper limits of agreement and their respective 95% confidence intervals (the former in red and the latter in green). The blue shading displays the 95% confidence interval built around the mean. For each figure, the ICC is also computed and displayed.

Then, a comparison of the averages of FEV_1_, FVC, and FEV_1_/FVC, expressed as a percentage of predicted values, was performed according to GLI 2012 and ERS 1993 reference values. Considering all 1355 workers, the FEV_1_ average was very similar between GLI 2012 and ERS 1993 (98.20% vs. 99.02%, *p* = 0.09), as well as for FVC (96.70% vs. 97.20%, *p* = 0.48), while for the FEV_1_/FVC ratio (101.22% vs. 102.64%, *p* < 0.0001) the GLI 2012 equations were closer to 100% of predicted values in a statistically significant way. In this regard, the agreement between GLI 2012 and ERS 1993 was analyzed using Bland–Altman plot and the ICC, comparing the different values of FEV_1_, FVC, and FEV_1_/FVC. The results of this comparison, shown in the Figure 1, indicate a small difference in the agreement of the two reference values. For the FEV_1_ measure, the lower limit of agreement (LLOA) was −5.20 (95% C.I. −5.48; −4.92) while the upper limit of agreement (ULOA) was 6.85 (95% C.I. 6.57; 7.13) and the ICC was 0.961. For the FVC measure, the LLOA was −8.60 (95% C.I. −9.02; −8.18) and the ULOA was 9.59 (95% C.I. 9.17; 10.01) with the ICC equal to 0.915. Finally, as regard the FEV_1_/FVC measure, the LLOA was −1.41 (95% C.I. −1.54; −1.28) and the ULOA was 4.26 (95% C.I. 4.12; 4.39) with an ICC of 0.946.

## 4. Discussion

The choice of the correct reference values is essential in spirometry. In this regard, considering that spirometric testing is an integral part of many health surveillance protocols, the availability of appropriate and adequate reference values for a correct interpretation of spirometry in workers is also essential in the evaluation of lung function in OM. The purpose of the reference equations is to provide accurate predictive values and LLNs to allow a clinical diagnosis. As stated by the 2005 ATS/ERS standards, for Europe no set of equations is recommended and the ERS 1993 reference equations are currently the most used in Italy and Europe. However, these equations are characterized by several limitations that complicate their use and have led to considerable criticism from the international scientific community [4,19]. Therefore, considering these limits, the 2005 ATS/ERS standards did not recommend the use of these reference equations and it is noteworthy to point out that recently the ERS/ATS technical standard on interpretive strategies for routine lung function tests clearly endorsed the GLI reference values stating that the GLI equations are the most generalizable suite of equations to date and that the predicted GLI values are consistently higher than ECSC (Table S1) [7,18,19,20,21]. Indeed, these reference values use a more recent and much more suitable mathematical model for describing changes in lung function that is the lambda-mu-sigma method. In this context, the aim of our study was to validate the GLI 2012 reference values in a healthy population of Italian workers. The experimental design of the study and the results obtained are in good agreement with the scientific literature available on this topic, i.e., validation studies for populations of different nations conducted on non-smoking Caucasian subjects, free from respiratory diseases. The validation studies taken as reference models are those carried out in Norway [11], Australia and New Zealand [12], Sweden [16], France [9], and Finland [15]. The chosen sample, as in all validation studies carried out to date, included subjects free of respiratory system diseases or other conditions that may affect the functionality of the respiratory system and non-smokers. The spirometric tests included in our analyses were all performed by workers exposed to chemical and/or biological risk factors. Although we are aware that such occupational exposures could negatively affect the pulmonary functionality, thus inducing both obstructive and possible restrictive patterns at the spirometric examinations, we feel the strict exclusion criteria adopted to select the analyzed tests in our study have allowed us to overcome this possible “sample selection bias”. In fact, all the subjects had a negative history of respiratory diseases and no documented alterations in pulmonary functionality at the time of the performance of the spirometric tests, and can reasonably considered a healthy population. Moreover, previously published validation studies did not elucidate possible occupational exposures in the enrolled populations, thus preventing the certain exclusion of such a possible workplace exposure impact on the lung functionality.

Given that the z-score should ideally be equal to 0 and consequently the SD equal to 0.1, this means that, in a population free of pathologies and non-smokers, the predicted FEV_1_, FVC, and FEV_1_/ FVC ratio is 100%. In our study, when the reference values GLI 2012 were applied, almost all 1355 workers had normal spirometry, with no obstructive deficits and only 58 (4.3%) showed a restrictive pattern. Importantly, no spirometry, for the FEV_1_/FVC ratio analyzed, had a z-score < −1.64, which represents the fifth percentile and is therefore considered the LLN. The ideal 0 was even closer when considering only the 719 normal-weight workers. Considering the gender, in our sample there was a statistically significant difference in the z-score averages between men and women for the three spirometric parameters studied. This further highlights the importance of the equations applied by the GLI 2012, as the anatomical and physiological differences were not highlighted by the linear regression equations applied by the ERS 1993. This result was also highlighted by the Finnish and Swedish validation studies [15,16].

Considering the BMI, our findings showed that, in obese subjects, the FVC and the FEV_1_/FVC ratio deviate from the ideal z-score of 0 in a statistically significant way and this finding is probably due to the onset of a possible restrictive pattern that is typical in these subjects. The age dependent trend of lung function has been highlighted by our study as much as in the Norwegian [11], Australian–New Zealand [12], Swedish, and Finnish studies [15,16]. In particular, in our study, considering the z-score averages for each age group, it was possible to notice that the middle ages (30−50 years) had the greatest proximity to 0 for FEV_1_. As for the FVC, there was instead a fairly linear increase in the distance from the ideal 0 with increasing age and similar results were obtained for the FEV_1_/FVC ratio. Therefore, the three spirometric indices analyzed did not follow a common linearity but have a distinct behavior in line with the trend of lung function described in the literature and with validation studies taken as a reference model [11,12,15,16].

Considering the z-score averages based on the height groups, the trend was similar for FEV_1_ and FVC. In this regard, shorter workers had an average z-score close to 0 and the workers taller than 165 cm had an average z-score further away from the ideal 0, while for the FEV_1_/FVC ratio the averages were instead similar in the three age groups. In this regard, the ANZSR study and the Norwegian study underline how important the approximation up to the first decimal digit is in detecting the height parameter [11,12]. Indeed, using the GLI 2012 equations, it was observed that the errors deriving from the self-reported height correspond to 1% and this affects the values of FEV_1_ and FVC by about 2.1% and 2.4%. Conversely, an opposite trend occurred in the Swedish and Norwegian populations where the underestimation of lung volumes was greater for subjects of short stature and lower for taller ones [11,16]. Obviously, these differences may be explained taking into account the different reference sample and its intrinsic anthropometric characteristics. In this regard, for example, Kainu et al., comparing their findings with those of Ben Saad et al., explained the discrepancy in these results based on the fact that the Tunisian population (which is part of the Caucasian population) had a significantly lower average height than the Norwegian population [14,15].

Finally, a comparison was made between the GLI 2012 and ERS 1993 equations in our Italian sample population. As regards FEV_1_, the average was very similar using GLI 2012 or ERS 1993, as well as for FVC, while for the FEV_1_/FVC the GLI 2012 obtained a statistically significant greater proximity to 100% of the predicted. Furthermore, Bland–Altman plot and ICC showed a small difference in the agreement of the two reference values. The differences highlighted by the comparison are due to the use, by the ERS 1993, of linear models, where it is assumed that the residual values are identical for each age and height. The comparison between ERS 1993 and GLI 2012 was also performed in the other studies. In Finland, an underestimation of 0.4 L was demonstrated for FEV_1_ and FVC, applying the ERS 1993 compared with the GLI 2012 with an even greater underestimation in women (527 mL equal to 17%) [15]. The superiority of these reference values, compared with the ERS 1993, is further underlined also in Sweden and Norway [11,16]. Interestingly, in these countries a comparison was also made with the local reference values, which are currently closer to 100% of the predicted compared with the GLI 2012, as they take into consideration exclusively the characteristics of the native population [11,16]. Furthermore, the French study showed that the z-scores for FEV_1_, FVC, and FEV_1_/FVC are closer to 0 than the ERS 1993 [9]. In addition, except for the FEV_1_/FVC ratio, the distribution of values below the LLN and above the Upper Limit of Normal are further away from the fifth percentile using the ERS equations (especially for women) [9]. In support of this thesis, the authors compared subjects who had values below the LLN (z-score < −1.64), applying the ERS and GLI equations and this comparison showed that the GLI reference equations better identified a possible respiratory disease compared to the ERS equations.

Our study presented some limitations. First of all, the instrument used to obtain the pulmonary function values was a portable spirometer, but in this regard it is useful to underline that this tool is the one commonly used in the daily practice of health surveillance carried out by the OPs. In addition, it should be pointed out that patient compliance could be a potential obstacle to a correct execution of spirometry test, but we minimized this limitation by including in the study only those spirometries that met the criteria of acceptability, usability, and repeatability set by ATS/ERS 2019 standards. Finally, it was a single-center study, which focused attention on a specific category of workers in a single city in southern Italy.

## 5. Conclusions

Our study showed that the GLI 2012 reference values are applicable to our Italian workers sample population, resulting as more accurate than the ERS 1993, especially in women, in normal-weighted subjects, in the middle-aged groups (30–50 years), and for height <165 cm. Furthermore, the GLI 2012, compared with the ERS 1993, providing a more adequately representative estimate of respiratory function and physiological variations with age, could be a more effective tool in diagnosing a possible obstructive deficit or restrictive pattern, as well as their severity. The comparison of the reference values showed that, for the GLI 2012, the FEV_1_/FVC ratio is on average 1.42% lower than the ERS 1993. Therefore, given the key role of this relationship in formulating the spirometric diagnosis, an incorrect interpretation of the spirometry test could occur since, for values close to the LLN, the uncritical use of ERS, is correlated to a tendency to underestimate a possible obstructive pathology in the initial phase.

Overall, our findings underlined the importance of choosing the most adequate reference values for spirometry in order to have a correct interpretative strategy. Although further studies are necessary to confirm this preliminary evidence, the results obtained allow us to suggest the application of the GLI 2012 reference values for the Italian working population that, depending on exposure to specific occupational risk factors, undergoes spirometry as part of the health surveillance program.

## Figures and Tables

**Table 1:** Main socio-demographics characteristics of population sample.

	Total Sample (*n* = 1355)	Men (*n* = 666)	Women (*n* = 689)
Gender	666 men (49.2%) 689 women (50.8%)		
Mean age (ys ± SD)	40.5 ± 15.3	45.7 ± 15.0	35.5 ± 13.9
Mean body height (cm ± SD)	168.6 ± 8.8	174.4 ± 7.1	163.1 ± 6.3
Mean body weight (kg ± SD)	73.9 ± 18.9	82.8 ± 17.0	65.3 ± 16.6
BMI (kg/m^2^ ± SD)	25.9 ± 5.8	27.2 ± 5.2	24.6 ± 6.1
Obesity	1129 non obese (83.3%) 226 obese (16.7%) 719 normal-weighted (53.1%) 410 overweight (30.3%) 134 obese class I (9.9%) 42 obese class II (3.1%) 50 obese class III (3.7%)	527 non obese (79.1%) 139 obese (20.9%) 245 normal-weighted (36.8%) 282overweight (42.3%) 97 obese class I (14.6%) 18 obese class II (2.7%) 24 obese class III (3.6%)	602 non obese (87.4%) 87 obese (12.6%) 474 normal-weighted (68.8%) 128 overweight (18.6%) 37 obese class I (5.4%) 24 obese class II (3.5%) 26 obese class III (3.8%)
Mean FEV_1_ (L ± SD)	3.40 ± 0.74	3.77 ± 0.75	3.06 ± 0.53
Mean FVC (L ± SD)	4.10 ± 0.89	4.60 ± 0.87	3.62 ± 0.60

**Table 2:** Spirometric parameters according to GLI 2012.

	Total Sample (*n* = 1355)			Normal-Weighted (*n* = 719)		
	Total	Men	Women	Total	Men	Women
Mean FEV_1_ (z-score and %)	−0.13 (98.20%)	−0.18 (97.55%)	−0.08 (98.82%)	−0.09 (98.85%)	−0.10 (98.62%)	−0.08 (98.96%)
Mean FVC (z-score and %)	−0.25 (96.78%)	−0.34 (95.40%)	−0.16 (97.96%)	−0.15 (98.19%)	−0.18 (97.73%)	−0.13 (98.43%)
Mean FEV_1_/FVC (z-score and %)	0.18 (101.22%)	0.29 (102.07%)	0.08 (100.40%)	0.07 (100.26%)	0.11 (100.61%)	0.05 (100.08%)

**Table 3:** Spirometric parameters using GLI 2012 according to the age.

	18–30 years	30–40 years	40–50 years	50–60 years	60–70 years	*p*-Value
Mean FEV_1_ (z-score and %)	−0.14 (98.28)	−0.03 (99.48)	−0.06 (99.07)	−0.17 (97.53)	−0.20 (96.74)	0.226
Mean FVC (z-score and %)	−0.16 (98.12)	−0.16 (98.08)	−0.22 (97.20)	−0.41 (94.39)	−0.38 (94.29)	<0.001
Mean FEV_1_/FVC (z-score and %)	−0.01 (99.69)	0.19 (101.09)	0.24 (101.56)	0.41 (103.10)	0.29 (102.40)	<0.001

**Table 4:** Spirometric parameters using GLI 2012 according to the height.

	<165 cm	165−175 cm	>175 cm	*p*-Value
Mean FEV_1_ (z-score and %)	−0.02 (99.49%)	−0.17 (97.71%)	−0.19 (97.41%)	0.008
Mean FVC (z-score and %)	−0.13 (98.15%)	−0.30 (96.14%)	−0.31 (95.87%)	0.002
Mean FEV_1_/FVC (z-score and %)	0.16 (101.03%)	0.20 (101.35%)	0.19 (101.23%)	>0.05

## Data Availability

All data generated or analyzed during this study are included in this published article.

## Supplementary Materials

The following supporting information can be downloaded at: https://www.mdpi.com/article/10.3390/ijerph192215200/s1, Table S1: ERS 1993 and GLI 2012 reference equations.
