# Supplementary material for: Validation of the Global Lung Function Initiative 2012 Spirometry Reference Values in a Healthy Italian Working Population
Source: Int J Environ Res Public Health. 2022 Nov 17;19(22):15200. doi: 10.3390/ijerph192215200 (PMC9690579; doi:10.3390/ijerph192215200)
Supplement: Supplementary file 1 [file ijerph-19-15200-s001.zip › ijerph-1929178-supplementary.pdf]

Table S1: ERS 1993 and GLI 2012 reference equations

	Reference equation
**GLI 2012**	Y = a + b·H + c·A + age-spline + d_1·group + d_2 ·group·A
**ERS 1993**	
	**Male subjects**
**FVC**	5.76H - 0.026A - 4.34
**FEV_1**	4.30H - 0.029A - 2.49
**FEV_1/FVC**	-0.18A + 87.21
	**Female subjects**
**FVC**	4.43H - 0.026A -2.89
**FEV_1**	3.95H - 0.025A - 2.60
**FEV_1/FVC**	-0.19A + 89.10

In the GLI 2012 reference equation Y is dependent variable, H the standing height (cm), A the age (yr), a, b, c, d_1 and d_2 are coefficients which vary for each ethnic group, and spline is an age-specific contribution from the spline function. Group is a dummy variable with values 0 or 1 indicating ethnicity, where Caucasians are the reference. Any of Y, H or A may be log transformed [8]. In the ERS 1993 summary equations (related to adults aged 18–70 years) for FVC, FEV_1 and FEV_1/FVC H is the standing height (cm) and A the age [5].
